# The Cell Wall Lipid PDIM Contributes to Phagosomal Escape and Host Cell Exit of *Mycobacterium tuberculosis*

**DOI:** 10.1128/mBio.00148-17

**Published:** 2017-03-07

**Authors:** Jeff Quigley, V. Keith Hughitt, Carlos A. Velikovsky, Roy A. Mariuzza, Najib M. El-Sayed, Volker Briken

**Affiliations:** a Department of Cell Biology and Molecular Genetics, University of Maryland, College Park, Maryland, USA; b Center for Bioinformatics and Computational Biology, University of Maryland—College Park, Maryland, USA; c W. M. Keck Laboratory for Structural Biology, University of Maryland Institute for Bioscience and Biotechnology Research, Rockville, Maryland, USA; Max Planck Institute for Infection Biology

## Abstract

The cell wall of *Mycobacterium tuberculosis* is composed of unique lipids that are important for pathogenesis. Indeed, the first-ever genetic screen in *M. tuberculosis* identified genes involved in the biosynthesis and transport of the cell wall lipid PDIM (*p*hthiocerol *dim*ycocerosates) as crucial for the survival of *M. tuberculosis* in mice. Here we show evidence for a novel molecular mechanism of the PDIM-mediated virulence in *M. tuberculosis*. We characterized the DNA interaction and the regulon of Rv3167c, a transcriptional repressor that is involved in virulence regulation of *M. tuberculosis*, and discovered that it controls the PDIM operon. A loss-of-function genetic approach showed that PDIM levels directly correlate with the capacity of *M. tuberculosis* to escape the phagosome and induce host cell necrosis and macroautophagy. In conclusion, our study attributes a novel role of the cell wall lipid PDIM in intracellular host cell modulation, which is important for host cell exit and dissemination of *M. tuberculosis*.

## IMPORTANCE

*Mycobacterium tuberculosis* is a major human pathogen that has coevolved with its host for thousands of years. The complex and unique cell wall of *M. tuberculosis* contains the lipid PDIM (*p*hthiocerol *dim*ycocerosates), which is crucial for virulence of the bacterium, but its function is not well understood. Here we show that PDIM expression by *M. tuberculosis* is negatively regulated by a novel transcriptional repressor, Rv3167c. In addition, we discovered that the escape of *M. tuberculosis* from its intracellular vacuole was greatly augmented by the presence of PDIM. The increased release of *M. tuberculosis* into the cytosol led to increased host cell necrosis. The discovery of a link between the cell wall lipid PDIM and a major pathogenesis pathway of *M. tuberculosis* provides important insights into the molecular mechanisms of host cell manipulation by *M. tuberculosis*.

## INTRODUCTION

*Mycobacterium tuberculosis* is one of the most deadly human pathogens due to its ability to manipulate and evade host innate and acquired immune responses (1–6). These capacities evolved during the longstanding interaction of *M. tuberculosis* with humans, lasting an estimated 50,000 to 70,000 years (7). One of the mechanisms by which *M. tuberculosis* establishes a favorable intracellular environment is through the manipulation of host cell death pathways in infected cells (3, 8–10). The prevailing model is that *M. tuberculosis* inhibits apoptosis at early stages of the infection, favoring replication, but induces necrosis at later stages in order to exit the host cell (8, 9). Nevertheless, the molecular mechanisms by which *M. tuberculosis* accomplishes these tasks remain poorly understood.

One of the first identified virulence factors of *M. tuberculosis* is the surface glycolipid PDIM (*p*hthicerol *dim*ycocerosates), when a PDIM-deficient *M. tuberculosis* H37Rv mutant was shown to be attenuated in a guinea pig model of infection (11). Subsequent signature tagged-transposon mutagenesis studies identified insertions in the PDIM operon that resulted in a similar attenuation in the mouse model of infection (12, 13). Several roles for PDIM function in pathogenesis have been proposed (for review, see references 2 and 14), including macrophage invasion (15), masking of pathogen-associated molecular patterns (PAMPS) (16, 17), resistance to killing by nitric oxide (16, 18, 19), and preventing the recruitment of activated macrophages to the site of infection (17). Nevertheless, there is still a great deal of uncertainty about the molecular mechanisms of PDIM-mediated virulence regulation of *M. tuberculosis* (2).

It is now well established that during their intracellular life cycle, a fraction of *M. tuberculosis* bacteria escape the phagosome (20–22; see reference 23 for review). Thus, increasing numbers of bacteria can be found in the cytosol over time until a threshold is reached and the host cell undergoes necrosis, allowing for exit of *M. tuberculosis* (21, 24). The process of host cell necrosis induction involves potentially multiple bacterial effectors and host cell signaling pathways that are only beginning to be understood (3, 8–10). Recently, our group described a hypervirulent *M. tuberculosis* mutant resulting from the deletion of the gene *Rv3167c*, a putative member of the tetracycline repressor (TetR)-like family of transcriptional regulators (TFR). Infection of macrophages with the M. tuberculosis *Rv3167c* deletion (Δ*Rv3167c* mutant) strain resulted in a significant increase in phagosomal escape, autophagy, and host cell necrosis compared to wild-type *M. tuberculosis*-infected cells (25). However, we did not identify the Rv3167c-regulated genes, which are responsible for the observed phenotypes. In the present study, we further investigated the virulence regulation by Rv3167c and uncovered a novel molecular mechanism for the action of PDIM on the host cell.

## RESULTS

### Rv3167c functions as a typical TFR.

**FIG 1:**
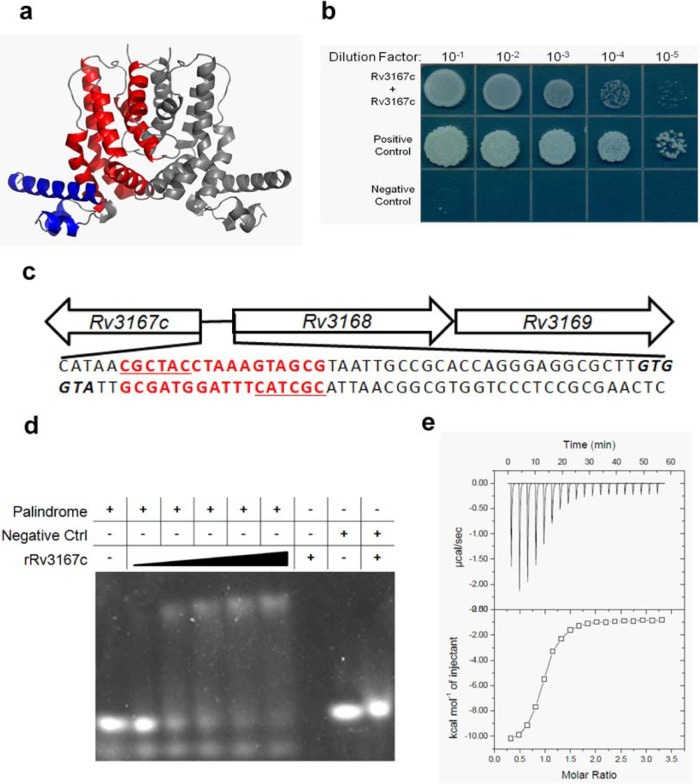
Rv3167c is a TetR-like transcriptional regulator. (a) Phyre^2^ predicted structure of Rv3167c modeled in PyMol. Helix-turn-helix DNA binding domain is colored blue. The ligand binding and dimerization domain is colored red. The second subunit of Rv3167c homodimer is colored gray. (b) Dilution series of *M. smegmatis* on trimethoprim agar coexpressing Rv3167c/Rv3167c fusion proteins and the positive-control interaction pair using the M-PFC system. (c) Organization of the *Rv3167c* genomic locus depicting the intergenic region between *Rv3167* and *Rv3168*. The putative palindromic Rv3167c binding site is in red (*palRv3167c*) with each arm of the palindrome underlined. Translational start of *Rv3167c* and *Rv3168* are in boldface and italic. (d) EMSA analysis of recombinant Rv3167c (rRv3167c) binding to *palRv3167c*. The rRv3167c concentration ranged from 2 to 30 μM. The *palRv3167c* concentration was constant at 200 nM. Randomized DNA serving as a negative control was used at 200 nM with 30 μM rRv3167c. The figure is representative of three independent experiments. (e) ITC analysis of rRv3167c binding to *palRv3167c*. The figure is representative of three independent experiments. The *K*_*d*_ (dissociation constant) of rRv3167c for *palRv3167c* is 12.3 ± 3.2 μM.

TFRs are among the most abundant family of transcriptional regulators in bacteria and as such have been extensively studied (26, 27). Rv3167c is annotated as a TFR since 89% of its protein sequence can be modeled by the Phyre2 software with 99.9% confidence using the highest-scoring template TFR (SCO0332) of *Streptomyces coelicolor*. The predicted structure depicts the N terminus containing 3 α-helices that form a typical helix-turn-helix DNA binding domain common to TFRs and the additional 6 α-helices that constitute the C-terminal ligand binding and dimerization domains (Fig. 1a). TFRs typically function as homodimers (26, 27). In order to test whether Rv3167c can dimerize, we made use of the mycobacterial protein fragment complementation (M-PFC) assay (28), where bacterial growth is seen on agar containing trimethoprim when the proteins of interest interact. As shown in Fig. 1b, growth was observed when Rv3167c was dually expressed, indicating that Rv3167c interacts with itself and likely forms a dimer. Depicted are the Rv3167c interaction, the positive control (GCN4, a yeast transcriptional regulator known to dimerize), and the negative control (empty plasmids). The full-length image, including all negative controls, can be found in Fig. S6 in the supplemental material. Additionally, we demonstrated that purified Rv3167c (molecular mass of 24 kDa) has a retention volume identical to that of ovalbumin (molecular mass of 43 kDa), indicating again that Rv3167c forms a homodimer (data not shown). TFRs are typically encoded in the genome in close proximity and in opposing orientation to the genes they regulate (29). Figure 1c depicts the genomic organization of the *Rv3167c* locus with the sequence of the intergenic region between *Rv3167c* and the adjacent operon, *Rv3168-Rv3169*. Based on transcriptome sequencing (RNA-seq) data, *Rv3168* and *Rv3169* are two of the most highly upregulated genes in the *M. tuberculosis* Δ*Rv3167c* mutant (see Fig. S1 in the supplemental material), suggesting direct regulation by Rv3167c.

10.1128/mBio.00148-17.1 FIG S1  Expression of *Rv3168* and *Rv3169*. Shown are RNA-seq mean quantile normalized read counts for (a) *Rv3168* and (b) *Rv3169* in wild-type *M. tuberculosis* (Mtb), the *M. tuberculosis* Δ*Rv3167c* deletion mutant, and the *M. tuberculosis* Δ*Rv3167c*::Comp complement strain. Download FIG S1, JPG file, 1 MB. Copyright © 2017 Quigley et al. 2017 Quigley et al. This content is distributed under the terms of the Creative Commons Attribution 4.0 International license.

### Rv3167c recognizes a binding site within the upstream region of its own promoter.

TFRs are frequently autoregulatory and bind to palindromic DNA sequences (26, 27). Interestingly, a palindrome (*palRv3167c*) exists within the *Rv3167c*-*Rv3168* intergenic region (sequence highlighted in red in Fig. 1c). Complementary oligonucleotides corresponding to both strands of *palRv3167c* were annealed to form a double-stranded product, which was incubated with purified Rv3167c and analyzed by electrophoresis mobility shift assay (EMSA). As shown in Fig. 1d, incubation of increasing concentrations of Rv3167c with *palRv3167c* resulted in an increased mobility shift indicating binding of Rv3167c to the sequence. Importantly, when Rv3167c was incubated with randomized double-stranded DNA of similar size, no shift was observed, demonstrating that Rv3167c’s interaction with *palRv3167c* is specific. We further characterized Rv3167c interaction with *palRv3167c* using isothermal titration calorimetry (ITC). Figure 1e is a representative example of three independent ITC experiments. Rv3167c bound *palRv3167c* in a 1:1 protein-DNA stoichiometry and had a dissociation constant of 12.3 ± 3.2 μM, which is similar to other TFR interactions with DNA. Importantly, Rv3167c demonstrated no significant interaction with randomized control DNA (see Fig. S2 in the supplemental material). Taken together, these data indicate that Rv3167c functions as a typical TFR that represses its own promoter and also negatively regulates the downstream gene(s)—in this case *Rv3168* and *Rv3169*.

10.1128/mBio.00148-17.2 FIG S2  Rv3167c does not bind randomized DNA probe. ITC was carried out as described in the Materials and Methods section to determine the degree of binding between rRv3167c and a randomized dsDNA probe. The figure is representative of two independent experiments. Download FIG S2, JPG file, 0.1 MB. Copyright © 2017 Quigley et al. 2017 Quigley et al. This content is distributed under the terms of the Creative Commons Attribution 4.0 International license.

### The Rv3167c regulon is enriched for genes involved in PDIM synthesis.

**FIG 2:**
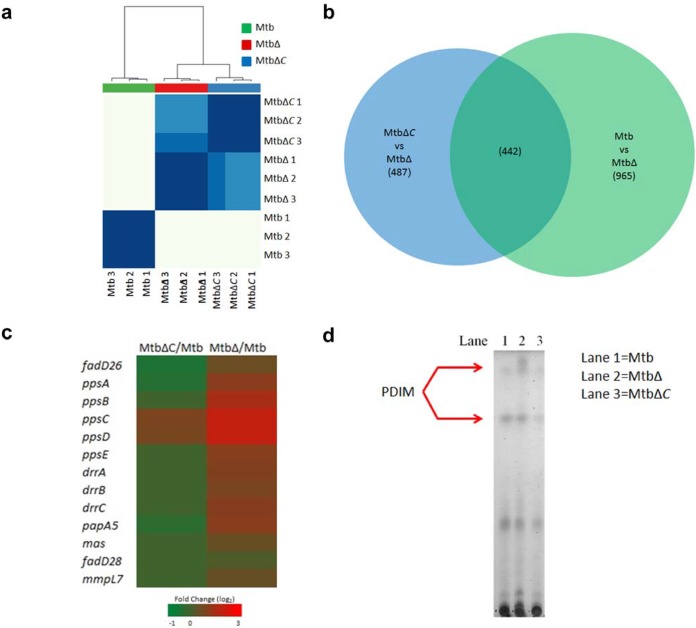
The TFR Rv3167c of *M. tuberculosis* regulates the PDIM gene cluster. RNA-seq analysis was performed on *in vitro*-grown cultures of wild-type *M tuberculosis*, the *M. tuberculosis* Δ*Rv3167c* deletion mutant (MtbΔ), and the *M. tuberculosis* Δ*Rv3167c* complemented (Δ*Rv3167c*::Comp) strain (MtbΔC) as shown (*n* = 3 per group). (a) Pearson correlation was used to determine similarities between samples followed by biclustering to generate a heat map depicting relationships between samples. The bottom and right axes are labeled with sample identifiers. The top axis depicts the relationship between sample groups. (b) Venn diagram depicting scheme to determine Rv3167c-regulated genes. Only genes in the overlap between Δ*Rv3167c* deletion mutant-*M. tuberculosis* and *M. tuberculosis* Δ*Rv3167c*::Comp*-*Δ*Rv3167c* mutant comparisons were considered. Numbers in parentheses indicate the number of genes deregulated. (c) Heat map depicting transcriptional fold changes of genes in the PDIM operon in the *M. tuberculosis* Δ*Rv3167c*::Comp*-*Δ*Rv3167c* mutant (first column) and Δ*Rv3167c* deletion mutant-*M. tuberculosis* (second column) comparisons. (d) TLC analysis of PDIM production by wild-type *M. tuberculosis*, the Δ*Rv3167c* mutant, and the Δ*Rv3167c*::Comp strain. Three hundred micrograms of total lipid was loaded in each lane. The plate was resolved in a mobile phase of 9:1 petroleum ether-diethyl ether. Lipid spots were revealed by charring.

In order to characterize the regulon of Rv3167c, we performed RNA-seq on the wild-type *M. tuberculosis*, *M. tuberculosis* Δ*Rv3167c* deletion mutant, and *M. tuberculosis* Δ*Rv3167c* complement (Δ*Rv3167*::Comp) strains grown in standard growth media and harvested during logarithmic growth. Our RNA-seq data were highly reproducible, as evidenced by the high degree of clustering between biological replicates, and indicated that we are analyzing distinct transcriptional states (Fig. 2a). The inclusion of the *M. tuberculosis* Δ*Rv3167*::Comp strain in our analysis provided a valuable internal control for determining the Rv3167c regulon. To do this, we determined genes differentially expressed in the *M. tuberculosis* Δ*Rv3167c* mutant compared to *M. tuberculosis* as well as genes differentially expressed in the *M. tuberculosis* Δ*Rv3167c* strain compared to the Δ*Rv3167c*::Comp strain. We compared both gene sets (Fig. 2b) and only considered genes differentially expressed in both contrasts as this gave us more confidence Rv3167c was involved in their regulation. In total, 442 genes were considered to be regulated by Rv3167c (see Table S1b in the supplemental material). The differential expression of such a number of genes indicates that Rv3167c has a relatively broad transcriptional impact. Gene Ontology (GO) enrichment analysis identified three GO terms (Table 1). The GO term “DIM/DIP cell wall layer assembly,” which encompasses genes associated with the synthesis and export of PDIM, was enriched in our gene set. Indeed, the entire PDIM operon was upregulated in the *M. tuberculosis* Δ*Rv3167c* mutant compared to wild-type *M. tuberculosis*, while being mostly unchanged or even downregulated in the *M. tuberculosis* Δ*Rv3167*::Comp strain compared to the wild type, indicating these genes do complement back to wild-type levels (Fig. 2c). Using thin-layer chromatography (TLC), we showed that the transcriptional upregulation manifested in an increase in PDIM lipid in the *M. tuberculosis* Δ*Rv3167c* mutant (Fig. 2d).

10.1128/mBio.00148-17.10 TABLE S1  (a) Primer and plasmid information. (b) List of differentially expressed genes in the *M. tuberculosis* Δ*Rv3167c* mutant. This is the list of the 442 genes that are deregulated in the wild-type *M. tuberculosis*-Δ*Rv3167c* mutant comparison (Mtb/MtbΔ) and Δ*Rv3167c*::Comp complement strain-Δ*Rv3167c* mutant comparison (MtbΔC/MtbΔ) shown in Fig. 2b. Download TABLE S1, XLSX file, 0.1 MB. Copyright © 2017 Quigley et al. 2017 Quigley et al. This content is distributed under the terms of the Creative Commons Attribution 4.0 International license.

**TABLE 1:** GO term enrichment of *M. tuberculosis* Δ*Rv3167c* mutant differentially regulated genes

Category	Term	No. of differentially expressed genes in category	No. of genes in category	Adjusted *P* value
GO:0071770	DIM/DIP cell wall layer assembly	9	9	0.0000062
GO:0006633	Fatty acid biosynthetic process	6	7	0.0048904
GO:0005618	Cell wall	123	659	0.0045409

### PDIM is involved in regulating host cell necrosis induction by *M. tuberculosis*.

**FIG 3:**
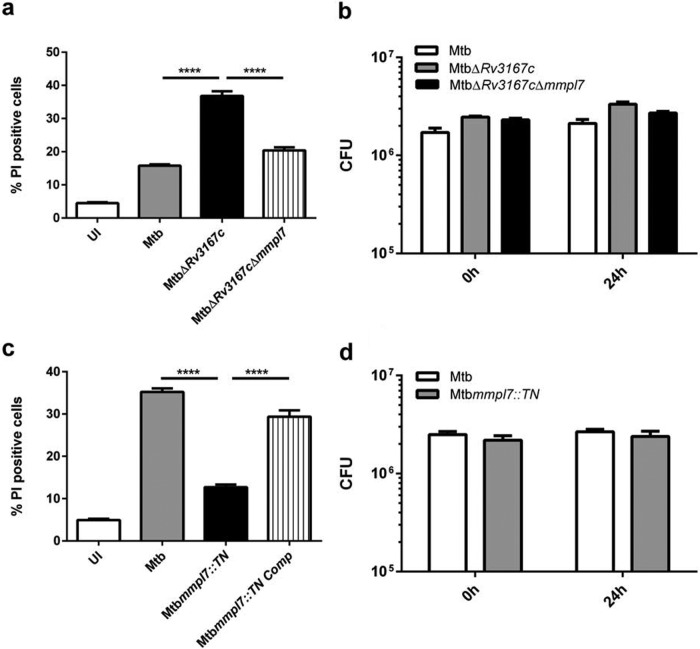
PDIM contributes to induction of host cell necrosis by *M. tuberculosis*. THP-1 cells were differentiated with PMA for 24 h and were left uninfected (UI) or were infected with the indicated *M. tuberculosis* strains. (a and c) Cells were harvested, stained with propidium iodide (PI), and analyzed by flow cytometry (*n* = 5,000) at 24 h postinfection. (b and d) Bacterial burden was determined by quantifying CFU immediately following infection (0 h) or 24 h postinfection (24 h). Data are representative of three independent experiments. Data are presented as means ± SEM. ****, *P* ≤ 0.0001 (one-way ANOVA).

PDIM is synthesized in the cytosol and transported to the outer membrane of *M. tuberculosis* by the membrane lipid transporter *mmpl7*. Deletion of *mmpl7* results in the failure of proper PDIM localization and the accumulation of the lipid in the cytosol (12). To assess the role of PDIM in the necrosis induced by the *M. tuberculosis* Δ*Rv3167c* mutant, we deleted *mmpl7* in the *M. tuberculosis* Δ*Rv3167c* background, resulting in the Δ*Rv3167c* Δ*mmpl7* double deletion mutant, which was confirmed by PCR (see Fig. S3 in the supplemental material). Infection of THP-1 macrophages with the *M. tuberculosis* Δ*Rv3167c* Δ*mmpl7* mutant resulted in a significant decrease in cell death compared to the Δ*Rv3167c* strain (Fig. 3a). As described above, RNA-seq analysis revealed more than 400 genes differentially regulated in the *M. tuberculosis* Δ*Rv3167c* strain. In an effort to minimize concerns about the contribution of other genes to the cell death phenotype and to assess whether PDIM alone was sufficient to modulate host cell death, we analyzed an *mmpl7* transposon insertion mutation (*mmpl7*::*TN*) in the *M. tuberculosis* CDC1551 strain obtained from BEI Resources (30). The deletion of *mmpl7* resulted in significantly less cell death than wild-type *M. tuberculosis* (Fig. 3c; see Fig. S9a in the supplemental material). Complementation of *mmpl7* back into the *M. tuberculosis mmpl7*::*TN* mutant reverted the phenotype to levels similar to those of the wild type (Fig. 3c). A clean deletion mutant generated through a recombination strategy (the *M. tuberculosis* Δ*mmpl7* mutant) confirmed this finding (see Fig. S7a in the supplemental material). Thin-layer chromatography confirmed that these mutants produce similar levels of PDIM to the wild type (see Fig. S8 in the supplemental material), confirming these mutants are deficient in export of PDIM, not synthesis. Previous work has implicated PDIM in macrophage invasion (15) and in resistance to killing by an early innate immune response (16, 18, 19). However, we saw similar numbers of bacteria both initially after infection and at 24 h postinfection in the *M. tuberculosis* Δ*Rv3167c*Δ*mmpl7* (Fig. 3b) and *mmpl7*::*TN* (Fig. 3d) mutants compared to wild-type *M. tuberculosis*. These data thus confirm that the bacterial PDIM levels directly impact the capacity of *M. tuberculosis* to induce host cell necrosis and do not just reflect a lower quantity of intracellular bacteria.

10.1128/mBio.00148-17.3 FIG S3  PCR confirmation of *mmpl7* knockout in the *M. tuberculosis* Δ*Rv3167c* Δ*mmpl7* double deletion mutant. The figure depicts the strategy and confirmation of the recombination of zeocin marker in place of *mmpl7*. Primer sets used to confirm knockout are labeled “LF” (left flank) and “RF” (right flank), which amplify the zeocin-gene junctions and only create a product if recombination was successful. The primer set labeled “IP” (internal priming) amplifies a region within *mmpl7* and will not create a product if recombination was successful. Genomic DNA was purified from the *M. tuberculosis* (Mtb) Δ*Rv3167c* and Δ*Rv3167c* Δ*mmpl7* mutants, and PCR was carried out with standard methods. Reactions were run on a 1.5% agarose gel. PCR product sizes are given in parentheses. Sequences of the primers used are given in Table S1. Download FIG S3, JPG file, 0.1 MB. Copyright © 2017 Quigley et al. 2017 Quigley et al. This content is distributed under the terms of the Creative Commons Attribution 4.0 International license.

### PDIM contributes to phagosomal escape of *M. tuberculosis* and host cell autophagy induction.

**FIG 4:**
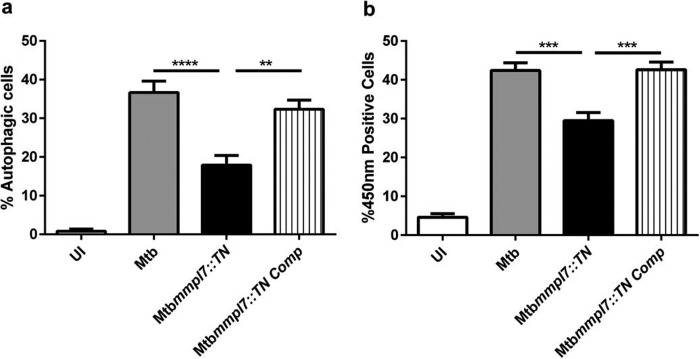
PDIM contributes to host cell autophagy induction and phagosomal escape of *M. tuberculosis*. (a) Differentiated THP-1 LC3-GFP-expressing cells were left uninfected (UI) or were infected with the indicated *M. tuberculosis* strains, and the cells were harvested 24 h later. The fraction of autophagic cells out of 5,000 total cells was determined by gating on GFP-positive cells after mild detergent treatment by flow cytometry as described. (b) The cleavage of CCF4-AM and increased fluorescence at 450 nm are an indicator for *M. tuberculosis* in the host cell cytosol. THP-1 cells were infected with the indicated strains for 24 h. Cells were then stained with CCF4-AM, and the number of cells emitting at 450 nm was determined by flow cytometry out of 5,000 total cells per condition. Data are representative of three independent experiments. Data are presented as means ± SEM. **, *P* ≤ 0.01; ***, *P* ≤ 0.001;****, *P* ≤ 0.0001 (one-way ANOVA).

Macroautophagy (autophagy) is an evolutionarily conserved catabolic process that involves sequestration of cytosolic contents into *de novo-*generated vesicles termed autophagosomes, which then get delivered to the lysosome for degradation. Intracellular pathogens can be targeted and killed by autophagic machinery in a process termed xenophagy (31). Previous work in our lab demonstrated that in addition to increased necrosis, infection of cells with the *M. tuberculosis* Δ*Rv3167c* mutant resulted in increased induction of autophagy but not xenophagy (25). In order to determine whether PDIM played a role in *M. tuberculosis*-induced autophagy in addition to the induction of necrosis, we monitored the conversion of cytosolic LC3I to autophagosome-bound LC3II, a hallmark of autophagy, using LC3-green fluorescent protein (GFP)-expressing THP-1 cells and flow cytometry (32). THP-1 LC3-GFP-expressing cells were infected for 24 h and partially lysed with saponin, and the remaining LC3-GFP was measured using flow cytometry (Fig. S9b for gating and raw data). A significant decrease in autophagy was seen in cells infected with the *M. tuberculosis mmpl7*::*TN* mutant compared to wild-type *M. tuberculosis* (Fig. 4a), a finding confirmed by analysis of the *M. tuberculosis* Δ*mmpl7* mutant (Fig. S7b) indicating PDIM plays a role in induction of autophagy in *M. tuberculosis*.

The *M. tuberculosis* Δ*Rv3167c* mutant accesses the cytosol earlier and to a greater extent than wild-type *M. tuberculosis* (25). We hypothesized that PDIM contributes to phagosomal escape and that loss of PDIM would result in decreased access to the cytosol. To test this, we made use of a fluorescence resonance energy transfer (FRET)-based assay described previously (21, 22). Briefly, THP-1 cells were loaded with CCF4-AM. Intact CCF4-AM emits green fluorescence (535 nm) due to FRET between the fluorescent moieties. Cleavage of CCF4-AM by β-lactamase expressed by cytosolic bacteria leads to FRET loss and a shift in the emission wavelength to 450 nm, which can be monitored by flow cytometry (Fig. S9c). Using this assay, we demonstrate that the *M. tuberculosis mmpl7*::*TN* mutant accesses the cytosol to a lesser extent than wild-type *M. tuberculosis* and the *mmpl7*::*TN* complemented strain (Fig. 4b). Previous work has shown that phagosomal escape of *M. tuberculosis* is dependent on the ESX-1-secreted substrate EsxA (33). We observed no difference in the secretion of EsxA in the *M. tuberculosis mmpl7*::*TN* mutant compared to wild-type *M. tuberculosis* (see Fig. S5 in the supplemental material). This represents the first evidence, to our knowledge, that PDIM influences the phagosomal escape of *M. tuberculosis*.

10.1128/mBio.00148-17.4 FIG S4  Knockout confirmation of the *M. tuberculosis mmpl7*::*TN* mutant. The transposon insertion site is marked with a red arrow. RNA was collected from each strain grown in 7H9 to an OD_600_ of 0.8, and cDNA was synthesized as described in Materials and Methods. The presence of *mmpl7* transcript was determined by PCR. The IP (internal priming) primer set amplifies a region within *mmpl7* downstream of the transposon insertion site. No reverse transcriptase (RT) was used to control for genomic DNA contamination. To confirm the presence and quality of RNA in the *M. tuberculosis* (Mtb) *mmpl7*::*TN* mutant, *sigA* transcript was amplified. Download FIG S4, JPG file, 0.1 MB. Copyright © 2017 Quigley et al. 2017 Quigley et al. This content is distributed under the terms of the Creative Commons Attribution 4.0 International license.

10.1128/mBio.00148-17.5 FIG S5  EsxA secretion is maintained in the *M. tuberculosis mmpl7*::*TN* mutant. Wild-type *M. tuberculosis* (Mtb), the *M. tuberculosis mmpl7*::*TN* mutant, and the *M. tuberculosis mmpl7*::*TN* Comp complement strain were grown in 50 ml Sauton’s media to an OD_600_ of 0.7. Bacteria were pelleted, and the supernatant was removed, filtered, and concentrated (culture filtrate). The bacterial pellet was lysed by bead beating and filtered (cell lysate). Ten micrograms of total protein was run on an anyKd SDS-PAGE gel (Bio-Rad), transferred to a polyvinylidene difluoride (PVDF) membrane (GE Healthcare), and blotted for EsxA, GroEL, or Fap. GroEL served as the lysis control. Fap served as loading control. EsxA antibody was used as 1:200, GroEL antibody was used at 1:50, and Fap antibody was used at 1:7,500. Download FIG S5, JPG file, 0.1 MB. Copyright © 2017 Quigley et al. 2017 Quigley et al. This content is distributed under the terms of the Creative Commons Attribution 4.0 International license.

10.1128/mBio.00148-17.6 FIG S6  M-PFC analysis of Rv3167c dimerization. Extended version of the M-PFC image depicted in Fig. 1b. Presented is a dilution series of *M. smegmatis* grown on trimethoprim agar coexpressing Rv3167c-Rv3167c fusion proteins from M-PFC-specific plasmids pUAB300 and pUAB400. The positive control is *M. smegmatis* coexpressing the yeast transcriptional regulator GCN4. Negative controls include expression of empty M-PFC plasmids as well as coexpression of each Rv3167c construct with the corresponding empty plasmid. Download FIG S6, JPG file, 0.1 MB. Copyright © 2017 Quigley et al. 2017 Quigley et al. This content is distributed under the terms of the Creative Commons Attribution 4.0 International license.

10.1128/mBio.00148-17.7 FIG S7  The *M. tuberculosis* Δ*mmpl7* mutant demonstrates decreased necrosis and autophagy. THP-1 cells were differentiated with PMA for 24 h and were left uninfected (UI) or were infected with the indicated bacterial strains. (a) Cells were harvested, stained with propidium iodide (PI), and analyzed by flow cytometry (*n* = 5,000) at 24 h postinfection. (b) Differentiated THP-1 LC3-GFP-expressing cells were left uninfected (UI) or were infected with the indicated strains, and the cells were harvested 24 h later. The fraction of autophagic cells out of 5,000 total cells was determined by gating on GFP-positive cells after mild detergent treatment by flow cytometry as described. (c) Bacterial burden was determined by quantifying CFU immediately following infection (0 h) or 24 h postinfection (24 h). Data are representative of three independent experiments. Data are presented as means ± SEM. ****, *P* ≤ 0.0001 (one-way ANOVA). (d) The panel depicts the strategy and confirmation of the recombination of the zeocin marker in place of *mmpl7*. Primer sets used to confirm knockout are labeled “LF” (left flank) and “RF” (right flank), which amplify the zeocin-gene junctions and only create a product if recombination was successful. The primer set labeled “IP” (internal priming) amplifies a region within *mmpl7* and will not create a product if recombination was successful. Genomic DNA was purified from wild-type *M. tuberculosis* (Mtb) and the Δ*mmpl7* mutant, and PCR was carried out by standard methods. Reactions were run on a 1.5% agarose gel. PCR product sizes are given in parentheses. The sequences of the primers used are given in Table S1. Download FIG S7, JPG file, 1.6 MB. Copyright © 2017 Quigley et al. 2017 Quigley et al. This content is distributed under the terms of the Creative Commons Attribution 4.0 International license.

10.1128/mBio.00148-17.8 FIG S8  The *M. tuberculosis mmpl7*::*TN* and Δ*mmpl7* mutants produce similar amounts of PDIM to wild-type *M. tuberculosis*. Shown are the results of TLC analysis of PDIM production by wild-type *M. tuberculosis* (Mtb) and the *mmpl7*::*TN* and Δ*mmpl7* mutants. Three hundred micrograms of total lipid was loaded in each lane. The plate was resolved in a mobile phase of 9:1 petroleum ether-diethyl ether. Lipid spots were revealed by charring. Download FIG S8, JPG file, 0.1 MB. Copyright © 2017 Quigley et al. 2017 Quigley et al. This content is distributed under the terms of the Creative Commons Attribution 4.0 International license.

## DISCUSSION

One of the goals of this study was to define the regulon of Rv3167c since we previously documented that this TFR represses virulence of *M. tuberculosis*, and hence its regulon should contain important virulence factors (25). Our RNA-seq analysis revealed a total of 442 genes differentially regulated in the *M. tuberculosis* Δ*Rv3167c* mutant suggestive of a broad transcriptional response. TFRs respond to environmental cues through their ligand binding domain (26, 27). We hypothesize that intracellular stressors such as low pH and increased reactive oxygen species (ROS) and nitrate species could be responsible for generating the Rv3167c ligand, but we failed to detect a stimulus that induces the loss of DNA binding by Rv3167c (data not shown). This is most likely due to the fact that *in vitro* approaches do not accurately replicate the complex milieu of a macrophage phagosome. The Rv3167c regulon is enriched for genes involved in the synthesis and transport of PDIM. There are several known transcriptional regulators of PDIM biosynthesis, such as *rpoB* (34) and *espR* (35), as well as posttranslational regulators, such as *pknH* (36). However, none of these genes is differentially regulated in the *M. tuberculosis* Δ*Rv3167c* mutant. The Rv3167c regulon contains several RNA polymerase sigma factors, including *sigE* and *sigB*, as well as 21 putative or known transcriptional regulators (Table S1). Differential expression of these regulators could explain the high degree of gene regulation we see in the *M. tuberculosis* Δ*Rv3167c* mutant and suggests that the control of PDIM gene expression by Rv3167c is indirect.

*M. tuberculosis* was long thought to reside exclusively within a vacuole that has the characteristics of an early endosomal compartment (1, 5, 37). However, it is now evident that *M. tuberculosis* also escapes the phagosome to reach the host cell cytosol (2, 38). After gaining access to the cytosol, *M. tuberculosis* induces necrosis in order to exit the infected cell and to disseminate (20, 21, 24, 39). Our previous findings indicated that the *Rv3167c* mutant consistently induced more necrosis and escaped the phagosome earlier and to a higher degree than wild-type *M. tuberculosis* (25). We hypothesized that the increased PDIM in the Δ*Rv3167c* mutant may play a role in the associated increase in necrosis induction and escape. This hypothesis was confirmed in the present study via deletion of the PDIM transporter *mmpl7* in the background of the *Rv3167c* mutant in *M. tuberculosis* H37Rv (Fig. 3a) and by using an *mmpl7* transposon mutation in the clinical strain CDC1551 (Fig. 3c). We propose that PDIM primarily contributes to the escape of *M. tuberculosis* from the phagosome and that the increase of host cell necrosis is merely a consequence of the increased number of cytosolic bacteria. However, it cannot be ruled out that PDIM has a direct role in induction of necrosis in addition to its function in phagosomal escape. Our previous findings demonstrated that induction of necrosis by the Δ*Rv3167c* mutant was dependent on increases in mitochondrial reactive oxygen species (25). *M. tuberculosis* lipids can traffic within endosomal membranes of host cells (40). While it has not been shown that *M. tuberculosis* lipids can also localize to host cell mitochondria, phagosomes do interact with mitochondria in order to optimize ROS production for defense against pathogens (41). Consequently, it is possible that lipids of *M. tuberculosis* inserted into the phagosomal membrane will be shared with mitochondrial membranes. PDIM may interact directly with mitochondrial membranes, leading to increased ROS production, which may initiate host cell necrosis.

The phagosomal escape of *M. tuberculosis* is dependent on the pore-forming activity of the ESX-1-secreted substrate EsxA (20, 22, 33). PDIM may influence *M. tuberculosis* escape through direct effects on EsxA secretion. Nevertheless, we show here that loss of PDIM does not affect secretion of EsxA (Fig. S5). Furthermore, while the deletion of *esxA* results in a complete lack of *M. tuberculosis* escape into the cytosol (20, 22, 33), the deletion of *mmpl7* leads to only an impaired escape (Fig. 4b). This suggests that while EsxA is absolutely necessary for *M. tuberculosis* to gain access to the cytosol, PDIM may work synergistically with EsxA to help *M. tuberculosis* escape the phagosome. Nevertheless, we are comparing these results with caution since the *esxA* mutant data were generated previously by a different lab and in a different genetic background. It was shown previously that EsxA favors association with liposomes containing phosphatidylcholine and cholesterol (42). While these lipids are not essential for insertion of EsxA (43, 44), the idea that EsxA may show preference for certain membrane compositions is enticing. Cholesterol is a critical component of mammalian cellular membranes as it provides the membrane rigidity necessary for signaling events (45). Certain biophysical properties, such as membrane fluidity, may promote insertion of EsxA into host membranes. PDIM has the ability to insert into host membranes, decreasing their fluidity (15). While not necessary for EsxA pore formation, insertion of PDIM into phagosomal membranes may alter the biophysical properties of the membrane to favor further EsxA insertion. There is also the possibility that EsxA and PDIM work independently toward the same goal. While EsxA is essential for gaining access to the host cell through membrane pore formation, PDIM may insert and weaken the phagosomal membrane, favoring bacterial escape.

The proposed role of PDIM in *M. tuberculosis* virulence is multifaceted (14), but there is still some uncertainty over what the molecular mechanisms are (2). For example, the impact of PDIM on cell entry seems to be marginal since it could only be demonstrated under very limited experimental conditions (15), and also our own experimental results did not detect any differences in uptake between wild-type *M. tuberculosis* and *mmpl7* mutants (Fig. 3b and d). PDIM is involved in the recruitment of activated macrophages to the site of infection in zebrafish (17). In light of our novel findings, one could hypothesize that the differential recruitment of macrophages may also be explained due to differences in host cell necrosis induction and dissemination mediated by wild-type *Mycobacterium marinum* versus the *mmpl7* mutant, which was not analyzed in the study (17). Indeed, the recruitment of neutrophils to infected areas of the lung is stimulated by the increase in host macrophage necrosis (46). Altogether, our findings establish a new and prominent role for PDIM in the phagosomal escape of and host cell necrosis induction by *M. tuberculosis*. This novel mechanism for manipulation of the host cell provides an additional answer for the long-standing puzzle of how PDIM contributes to the virulence of *M. tuberculosis*.

## MATERIALS AND METHODS

### Bacterial strains and growth conditions.

All *M. tuberculosis* cultures were grown at 37°C in 7H9 broth or on 7H11 agar medium with 1× ADC enrichment (albumin-dextrose-catalase), 0.5% glycerol, and 0.05% Tween 80 (broth). *Escherichia coli* cultures were grown in LB medium. Kanamycin, hygromycin, and zeocin were used at 40, 50, and 100 μg/ml, respectively, for *M. tuberculosis* cultures and 40, 150, and 100 μg/ml, respectively, for *E. coli*.

### Cell culture and infections.

THP-1 cells from ATCC were maintained at 37°C and 5% CO_2_ in RPMI (ATCC) supplemented with 10% heat-inactivated fetal calf serum (FCS) (growth medium). THP-1 LC3-GFP-expressing cells were grown in growth medium plus 100 μg/ml G418 sulfate (Cellgro). For infections, THP-1 cells were treated with 20 ng/ml phorbol 12-myristate 13-acetate (PMA) (Sigma) for 24 h to differentiate. Cells were washed twice in phosphate-buffered saline (PBS) and infected at a multiplicity of infection (MOI) of 3:1 in growth medium plus 5% human serum AB (Sigma). The infected cells were incubated at 37°C and 5% CO_2_ for 4 h followed by two more PBS washes and chased with growth medium plus 100 μg/ml gentamicin (Gibco). The time point indicated as “0 h” refers to immediately after 4 h of infection and washes, while “24 h” refers to the 24-h chase period after 4 h of infection.

### Cloning and construction of *M. tuberculosis* mutants.

DNA recombinant techniques were carried out following standard procedures. All restriction and modifying enzymes were purchased from Fermentas. *M. tuberculosis* mutants were constructed using a recombineering approach and specialized phage transduction as described previously (47). Knockouts were confirmed by PCR. Sequences of all primers used in this study can be found in Table S1. The following reagent was obtained through BEI Resources, NIAID, NIH: *Mycobacterium tuberculosis* strain CDC1551 (NR-13649), transposon mutant 1224 (MT3012/Rv2942/*mmpl7*: open reading frame [ORF] size, 2,763; point of insertion, 354; NR-14743). Complementation of the *mmpl7* transposon and deletion mutants was carried out by expression of full-length *mmpl7* from the episomal plasmid pMAN-1, kindly provided by Jeff Cox (48). Primers used for complementation can be found in Table S1a.

### Protein purification.

Rv3167c was cloned into pET28c (Novagen) using the BamHI and NdeI restriction sites to generate an N-terminal 6-histidine tag. The cloned construct was transformed into *E. coli* BL21-DE3. BL31-DE3::pET28c-Rv3167c was grown in LB plus kanamycin to an optical density at 600 nm (OD_600_) of 0.5, after which expression of Rv3167c was induced with the addition of 1 mM IPTG (isopropyl-β-d-thiogalactopyranoside) for 5 h. The culture was harvested, resuspended in PBS plus 200 mM NaCl, and disrupted by sonication. Cellular debris was removed by spinning at 15,000 × *g* for 30 min. The supernatant was applied to columns packed with His-Pur cobalt Superflow resin (Thermofisher) equilibrated with PBS plus 200 mM NaCl plus 10 mM imidazole and incubated at 4°C for 1 h with rocking. The column was washed with 10 volumes of PBS plus 200 mM NaCl plus 30 mM imidazole. Rv3167c was eluted from the column with PBS plus 200 mM NaCl plus 250 mM imidazole. Ten percent glycerol was added, and the purified protein was stored at 4°C until fast protein liquid chromatography (FPLC) purification.

### FPLC.

All FPLC experiments were carried out on a GE AKTA Explorer chromatography system. Gel filtration chromatography was carried out on a Superdex 75HR 10/30 column (GE Healthcare Life Sciences). Eluent was then further purified with ion-exchange chromatography on a MonoQ 5/50 Gl column (GE Healthcare Life Sciences). Purified protein was confirmed to be Rv3167c by mass spectrometry. An LMW gel filtration calibration kit (Amersham Biosciences) was used as the standard for gel filtration chromatography determination of Rv3167c dimerization.

### ITC.

Isothermal titration calorimetry (ITC) was performed on a MicroCal iTC200 titration microcalorimeter with a starting protein concentration of 0.130 mM and a starting double-stranded DNA (dsDNA) concentration of 2.10 mM. Immediately before the experiments were conducted, the protein was dialyzed against 10 mM phosphate (pH 7.2), 136 mM NaCl, and 4 mM KCl to remove glycerol. DNA oligonucleotides were annealed in the same buffer as the protein. Each titration experiment consisted of 2-μl injections of dsDNA into protein at 25°C with a mixing speed of 1000 rpm. Data acquisition and analysis were performed with the software package ORIGIN according to a single-site binding model.

### EMSA.

Two oligonucleotides per tested binding site were purchased from Operon, corresponding to the plus and minus strands of each site. To anneal, corresponding oligonucleotides were mixed at a 1:1 molar ratio and heated to 95°C for 10 min in a heat block, after which the block was turned off and allowed to slowly cool to room temperature. Annealed oligonucleotides were mixed with purified Rv3167c at the indicated concentrations in a buffer containing PBS plus 100 mM NaCl plus 5 mM MgCl_2_ and incubated on ice for 15 min. Samples were then separated on a 5% Tris-borate-EDTA (TBE) PAGE gel (Bio-Rad) at 150 V for 1 h at 4°C. The gel was then stained with SYBR green (Thermo, Fisher) and imaged. Randomized oligonucleotides served as the negative control. All primer sequences are listed in Table S1a.

### PI and LC3-GFP staining for cell death and autophagy analysis.

For propidium iodide (PI) staining, at the indicated time points, cells were harvested and washed once in PBS and then resuspended in PBS plus 5% FCS and 1 μg/ml PI and incubated for 10 min. The percentage of PI-positive cells was determined by flow cytometry (BD Accuri C6). Analysis of LC3-GFP-expressing THP-1 cells was performed as described previously (21). At the indicated time points, cells were harvested and washed once with PBS. Cells were then permeabilized with 0.05% saponin for 5 min, after which they were washed once in PBS and resuspended in PBS plus 5% FCS. The percentage of autophagic cells was determined by flow cytometry (BD Accuri C6). A representative example of the flow cytometry gating strategy to determine PI and LC3-GFP positivity can be found in Fig. S9a and b.

10.1128/mBio.00148-17.9 FIG S9  Representative example of the gating strategy used to quantify necrosis, autophagy, and phagosomal escape. (a) Necrosis was determined by propidium iodide (PI) staining as described in Materials and Methods. The left panel depicts the gating on healthy cells by forward scatter and side scatter. Uninfected (UI) sample is depicted. The right panel depicts analysis of PI positivity of healthy cells as measured by a shift to the right in the FL2 channel. Any cell to the right of the vertical gate (red vertical line) was considered PI positive. Black indicates uninfected, red indicates wild-type *M. tuberculosis*, blue indicates the *M. tuberculosis mmpl7*::*TN* mutant, and yellow indicates the *M. tuberculosis mmpl7*::*TN* Comp complement. (b) Autophagy was determined by the retention of LC3-GFP. The left panel depicts the gating on healthy cells by forward scatter and side scatter. Uninfected sample is depicted. The right panel depicts analysis of LC3-GFP retention in healthy cells as measured by a shift to the right in FL1 channel. Any cell to the right of the vertical gate (red vertical line) was considered LC3 positive, representative of an autophagic cell. Black indicates uninfected, red indicates wild-type *M. tuberculosis*, blue indicates the *M. tuberculosis mmpl7*::*TN* mutant, and yellow indicates the *M. tuberculosis mmpl7*::*TN* Comp complement. (c) To measure phagosomal escape, the loss of FRET from the molecule CCF4-AM was monitored. Healthy cells were first gated according to their forward and side scatter shown in the left panel to disregard cell debris. Next, LIVE/DEAD fixable red stain was used to gate on the live populations shown in the “live” gate. The uninfected population is depicted. Finally, live cells were monitored for an increase in 450-nm fluorescence indicating a loss of FRET. These cells are identified by a shift into gate Q2 in the two rightmost panels indicating high 450-nm fluorescence. Uninfected and wild-type populations are depicted. Download FIG S9, JPG file, 2.9 MB. Copyright © 2017 Quigley et al. 2017 Quigley et al. This content is distributed under the terms of the Creative Commons Attribution 4.0 International license.

### M-PFC.

Mycobacterial protein fragment complementation (M-PFC) was conducted as described in reference 28. Rv3167c was cloned into both pUAB300 and PUAB400. Constructs were then cotransformed into *Mycobacterium smegmatis*. Transformants were then spotted with appropriate controls on 7H11 medium supplemented with 50 μg/ml trimethoprim (Fisher) and grown at 37°C for 3 days.

### FRET assay for translocation of *M. tuberculosis* in host cell cytosol.

To detect mycobacterial escape from the phagosome, the CCF4 FRET assay was performed as described previously (21). Briefly, cells were stained with 8 μM CCF4 (Invitrogen) in EM buffer (120 mM NaCl, 7 mM KCl, 1.8 mM CaCl_2_, 0.8 mM MgCl_2_–5 mM glucose, 25 mM HEPES [pH 7.3]) containing 2.5 μM probenecid (Sigma-Aldrich) for 1.5 h at room temperature. Live populations were distinguished from dead ones by addition of LIVE/DEAD fixable red stain (Invitrogen) for 30 min at room temperature. Following staining, cells were fixed with 4% paraformaldehyde (PFA) overnight and analyzed by flow cytometry (BD FACSCanto). Representative examples of the flow cytometry gating strategy to determine loss of FRET in infected cells can be found in Fig. S9c.

### RNA isolation.

*M. tuberculosis* cultures were grown in 7H9 until they reached an OD_600_ of 0.6 to 0.8. Cultures were pelleted and resuspended in 1 ml TRIzol (Ambion). RNA was extracted with chloroform, precipitated with 100% isopropanol, and washed with 70% ethanol. Purified RNA was treated with Turbo DNase (Ambion) for 1 h. RNA was used right away or stored at −80°C.

### cDNA synthesis.

The Maxima first strand cDNA synthesis kit (Thermo Scientific) was used to synthesize cDNA as per the manufacturer’s instructions.

### RNA-seq library preparation and analysis.

Ribosomal RNA was removed from samples using the Epicentre Gram-positive Ribo-Zero rRNA magnetic removal kit. RNA-seq libraries were prepared using the Illumina ScriptSeq v2 library preparation kit according to the manufacturer’s protocol. Library quality was assayed by Bio-analyzer (Agilent) and quantified by quantitative PCR (qPCR) (Kapa Biosystems). Libraries were run on an Illumina HiSeq1500, generating 100-bp paired end reads. RNA-seq read quality was verified using FastQC, and low-quality base pairs were trimmed using Trimmomatic. The *M. tuberculosis* H37Rv reference genome was downloaded from TB Database, and reads were mapped to the genome using TopHat2. Count tables were generated using HTSeq along with a custom GFF, which includes features from both TB Database and Tuberculist. Count tables were loaded into R/Bioconductor, quantile normalized, and corrected for variance bias using Voom. Limma was used to find genes that were differentially expressed between each of the pairs of sample conditions. A total of 442 genes were found to be both differentially expressed in the wild-type–mutant contrast and the mutant-complement contrast and thus are potentially affected by Rv3167c regulation. This list of candidate deregulated genes was checked for functional enrichment using GOseq with multiple-testing correction using the Benjamini and Hochberg method (49).

### Total lipid extraction and TLC.

Cultures were grown in 7H9 broth in a volume of 50 ml to an OD_600_ of 0.8. Cultures were pelleted and resuspended in 2:1 methanol-chloroform and left at 4°C overnight. Samples were spun down, and supernatant was transferred to a glass beaker. The pellet was resuspended in 1:1 methanol-chloroform and left at room temperature for an hour. Samples were spun down, and the supernatant was removed. The process was repeated again with 1:2 methanol-chloroform. All three fractions were pooled, and the methanol and chloroform were allowed to evaporate overnight in a fume hood. The total lipid pellet was weighed, and 300 μg of total lipid was spotted onto a TLC plate. The plate was resolved in a mobile phase of 9:1 petroleum ether-diethyl ether. Lipid spots were revealed by spraying the plate with a solution of 3% cupric acetate in 8% phosphoric acid followed by baking at 140°C.

### Immunoblotting.

Bacteria were pelleted and resuspended in 25 mM Tris HCl, followed by bead beating to lyse cells. Lysate was cleared by spinning at 12,000 rpm for 5 min. The Pierce bicinchoninic acid (BCA) protein assay kit (Thermo Scientific) was used to measure protein concentrations. Antibody against EsxA was purchased from Santa Cruz Biotechnology, Inc., and used at a 1:200 dilution. Antibodies against Fap and GroEL were obtained from BEI Resources and used at 1:7,500 and 1:50 dilutions, respectively.

### Statistical analysis.

Statistical analysis was performed using GraphPad Prism version 6.0 software. Data are presented as means ± standard errors of the means (SEM) from three independent experiments, and one-way analysis of variance (ANOVA) with Tukey’s posttest was used unless mentioned otherwise in the figure legends. *P* value significance is indicated on the figures as follows: *, *P* ≤ 0.05; **, *P* ≤ 0.01; ***, *P* ≤ 0.001; and ****, *P* ≤ 0.0001.
