# Supplementary figures and images for: The Cell Wall Lipid PDIM Contributes to Phagosomal Escape and Host Cell Exit of Mycobacterium tuberculosis
Source: mBio. 2017 Mar 7;8(2):e00148-17. doi: 10.1128/mBio.00148-17 (PMC5340868; doi:10.1128/mBio.00148-17)

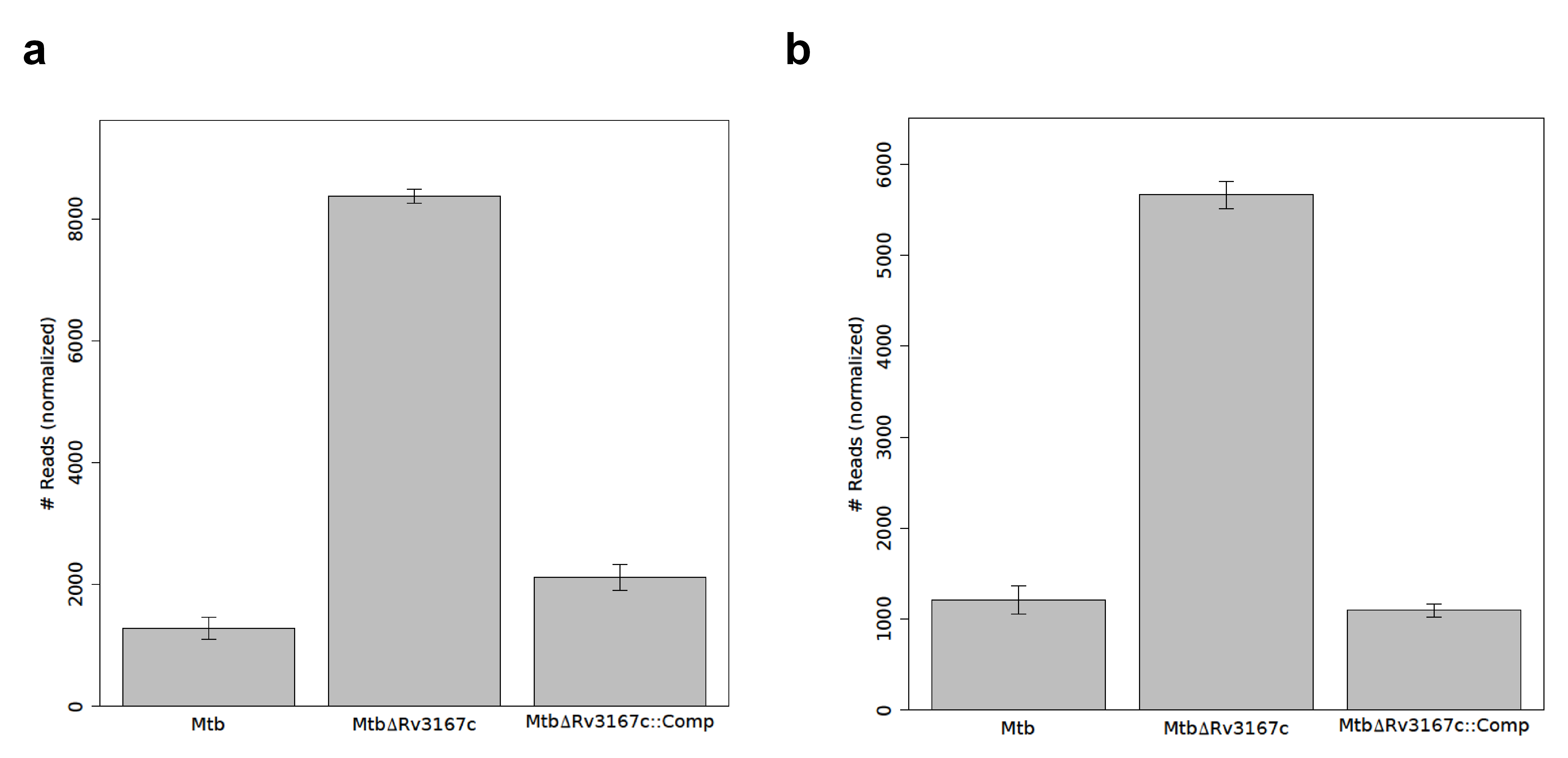

Supplement: FIG S1 [file mbo001173225sf1.jpg]

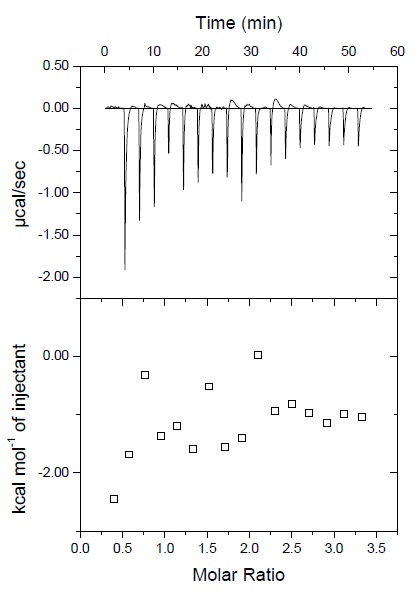

Supplement: FIG S2 [file mbo001173225sf2.jpg]

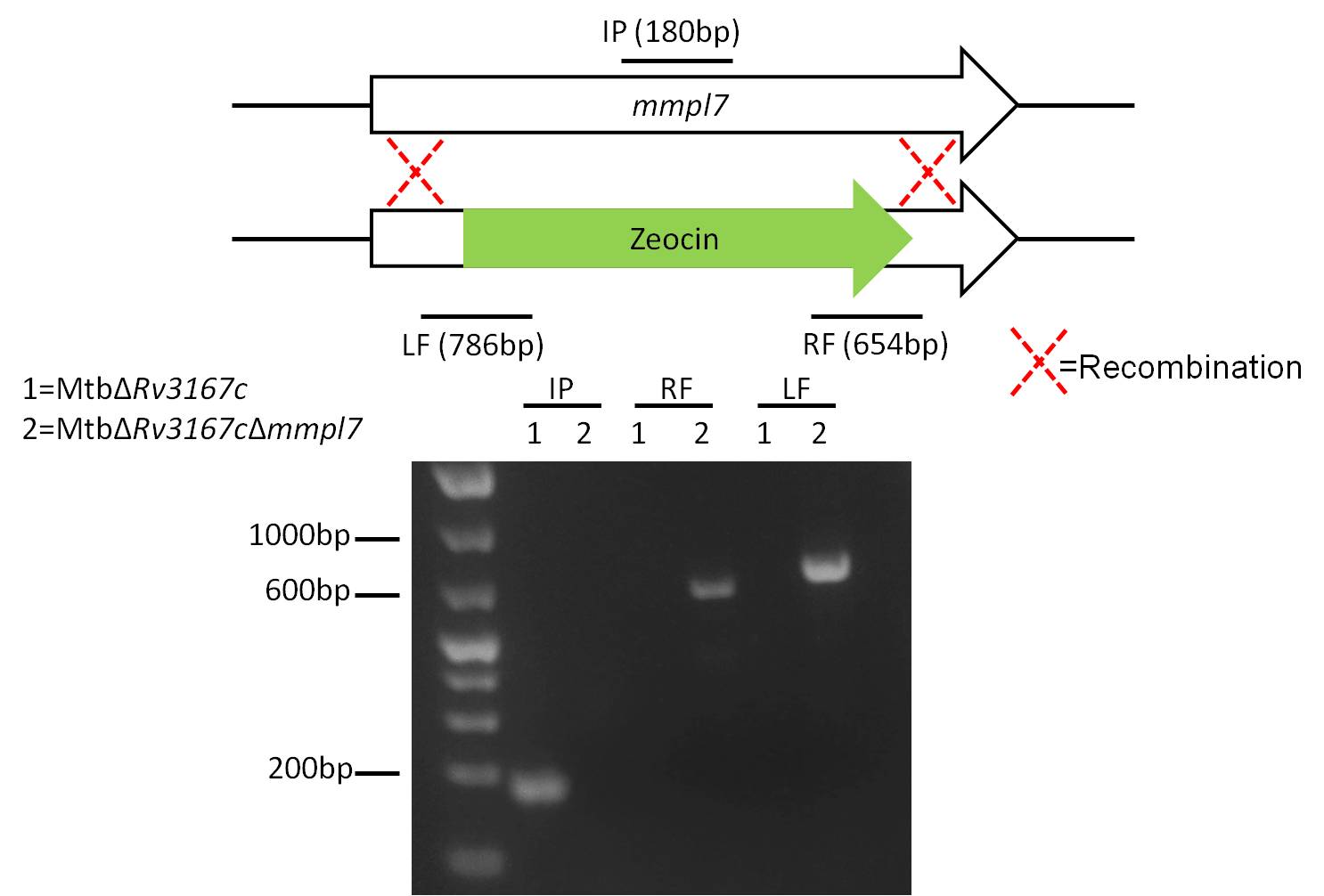

Supplement: FIG S3 [file mbo001173225sf3.jpg]

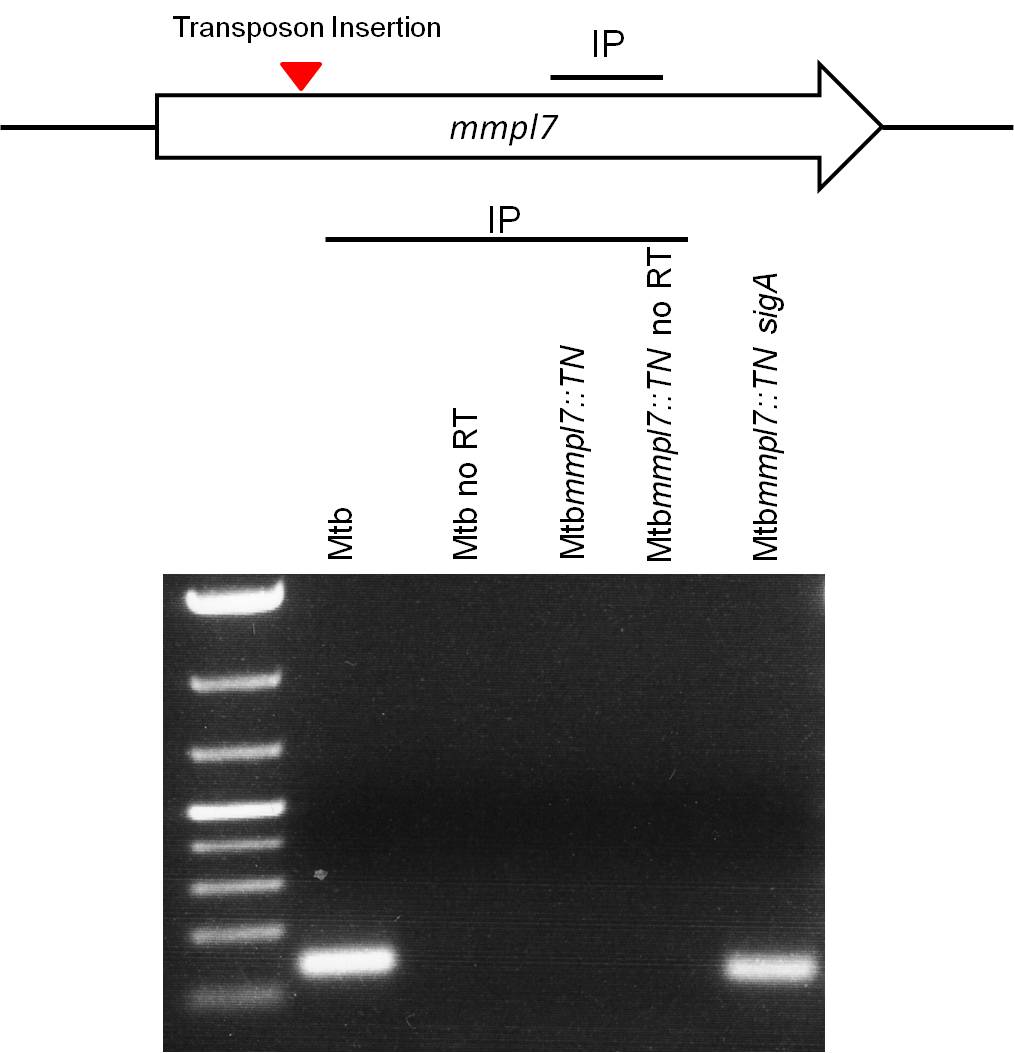

Supplement: FIG S4 [file mbo001173225sf4.jpg]

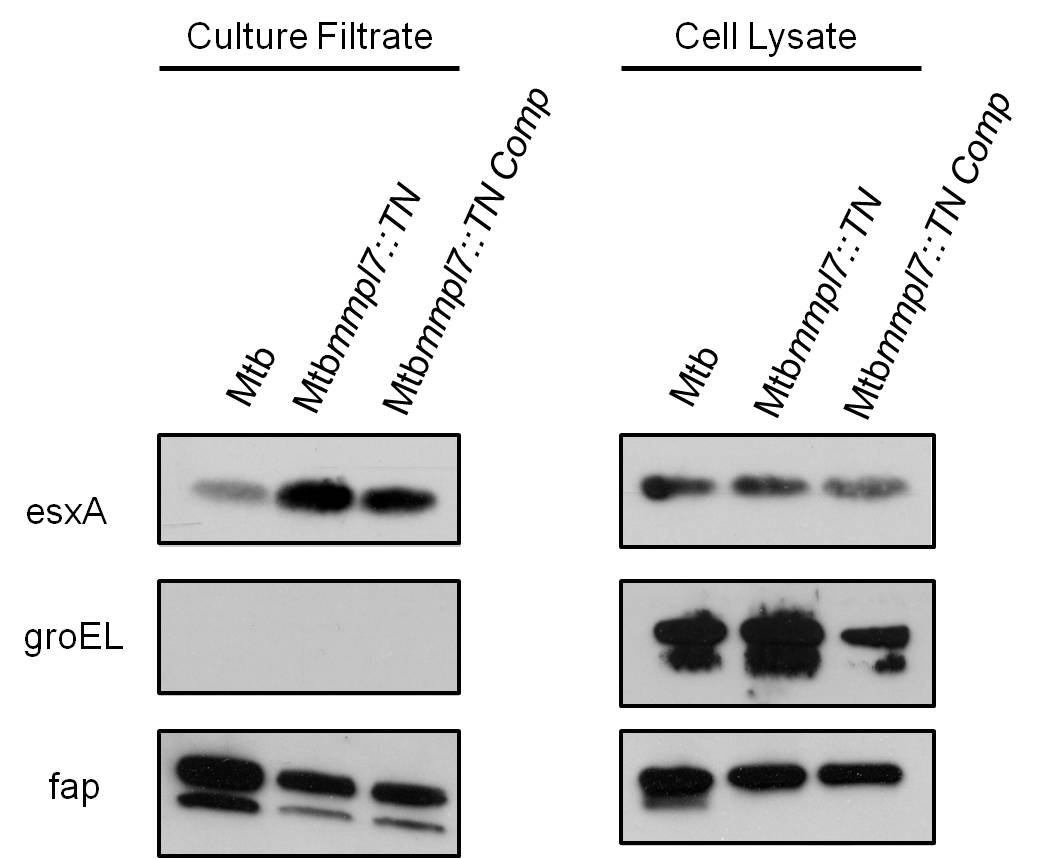

Supplement: FIG S5 [file mbo001173225sf5.jpg]

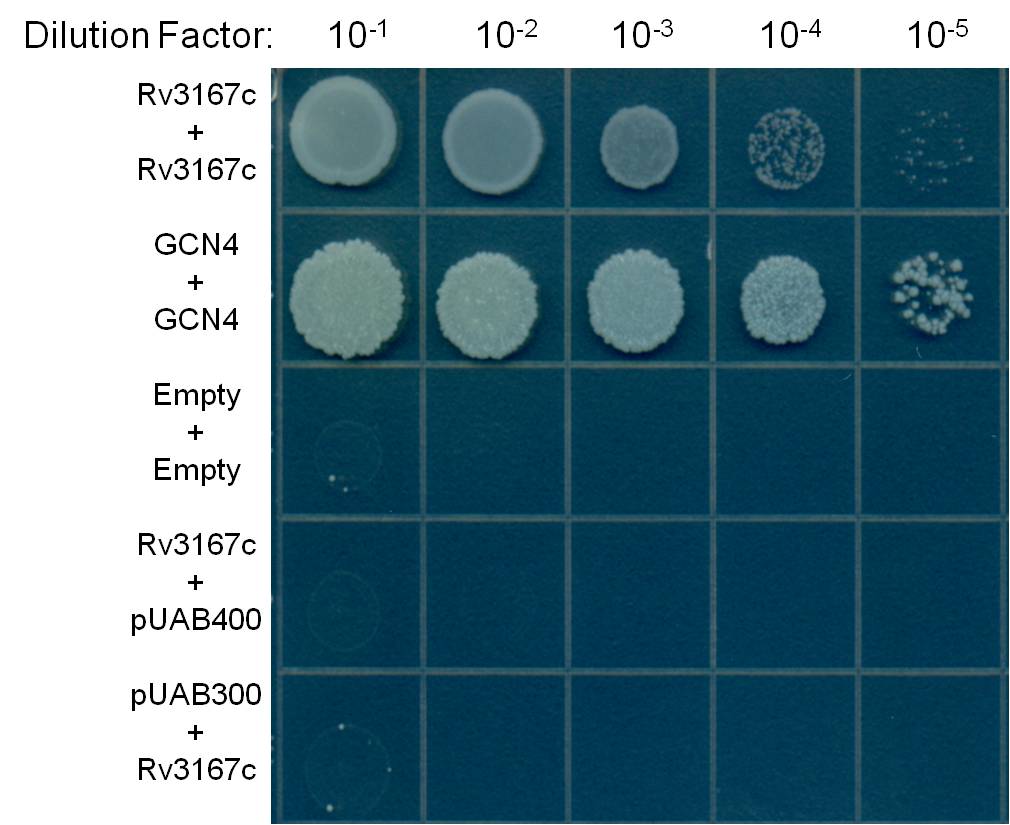

Supplement: FIG S6 [file mbo001173225sf6.jpg]

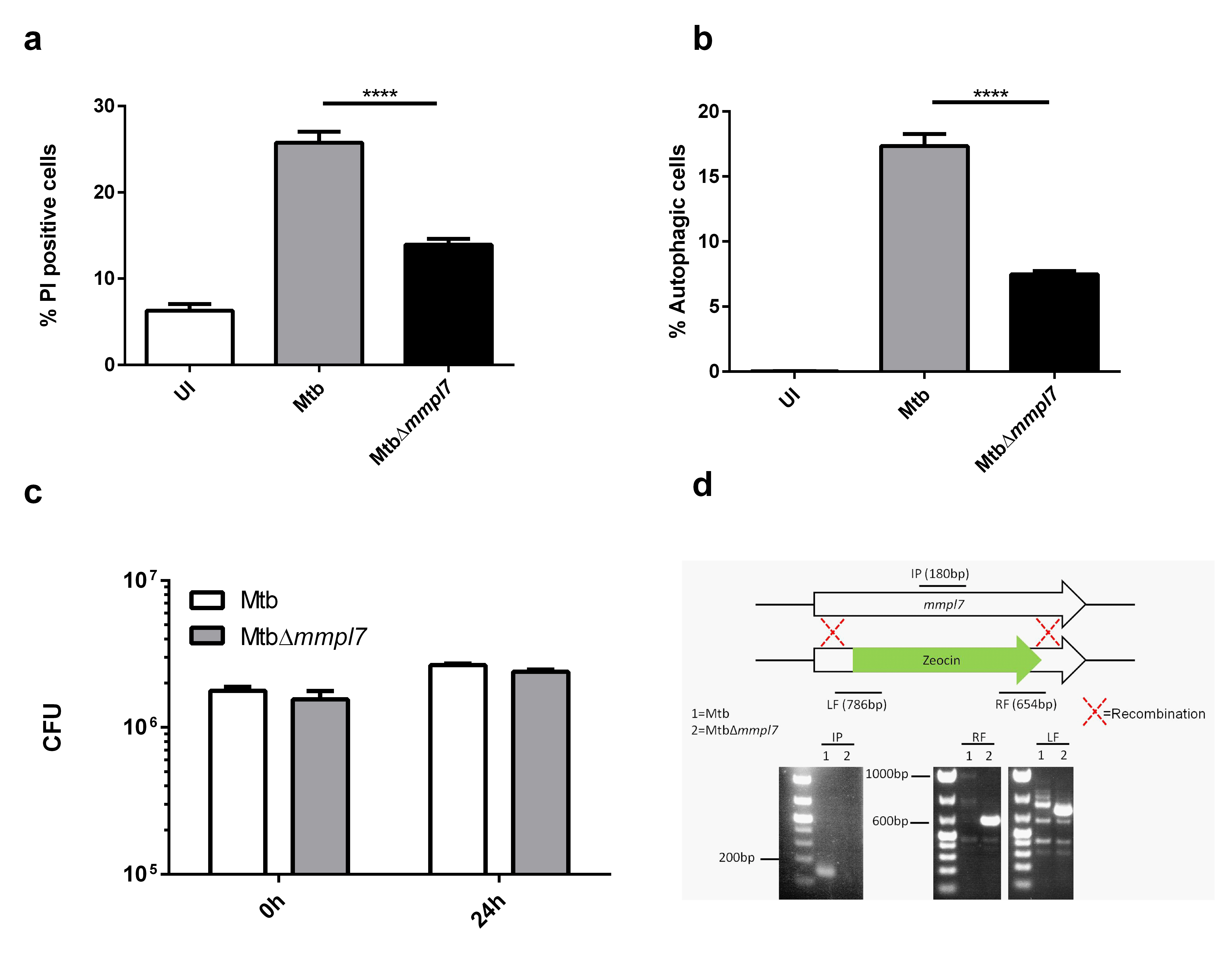

Supplement: FIG S7 [file mbo001173225sf7.jpg]

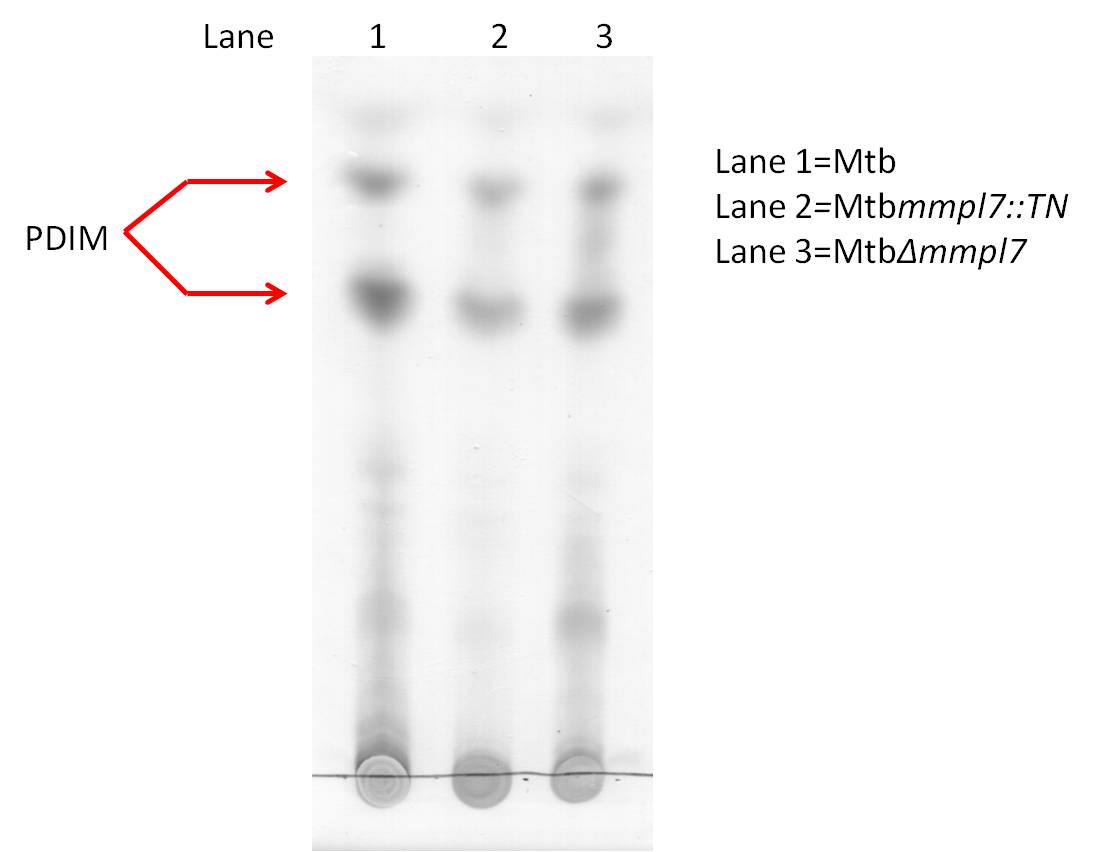

Supplement: FIG S8 [file mbo001173225sf8.jpg]

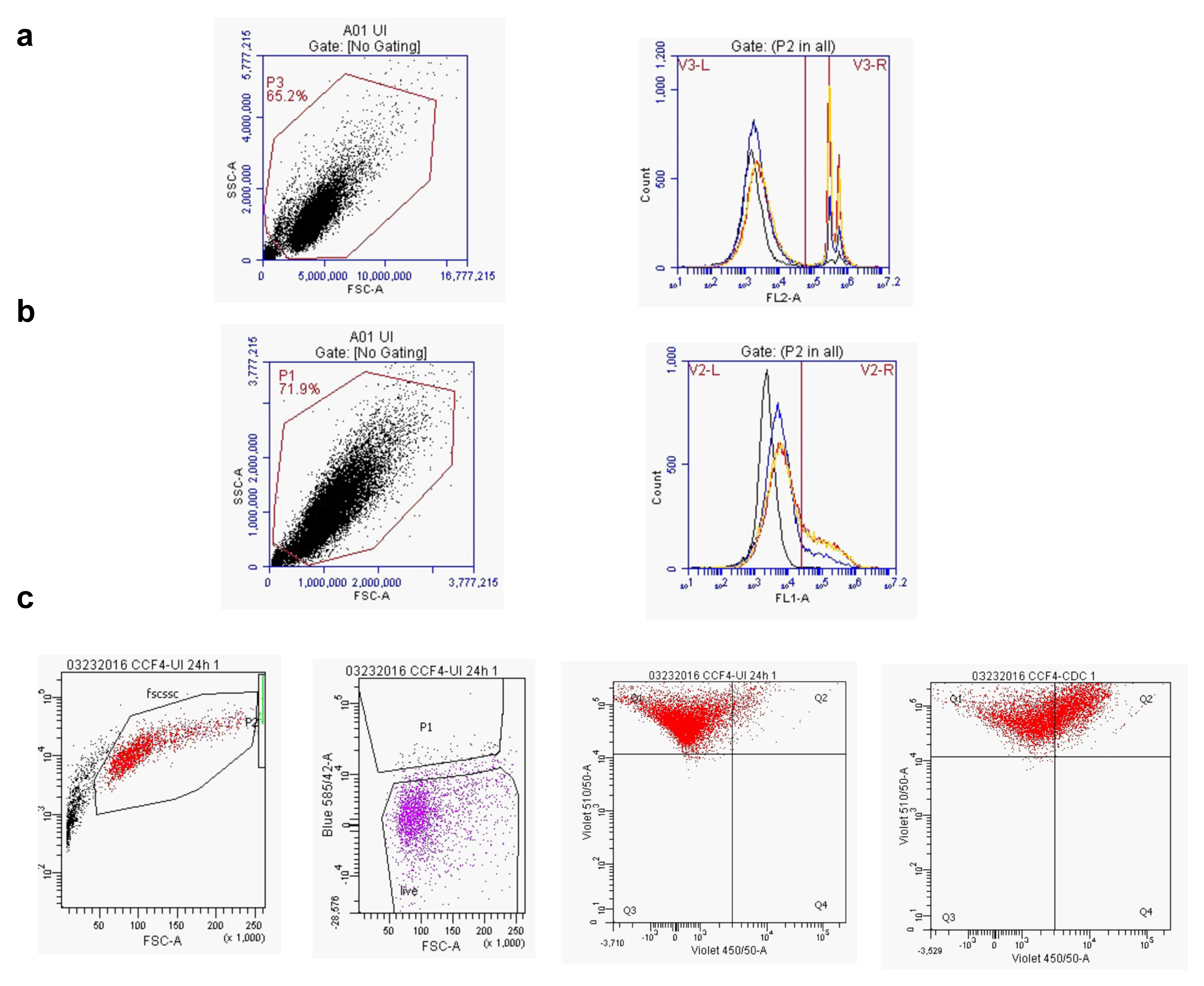

Supplement: FIG S9 [file mbo001173225sf9.jpg]
